# Potential application of oxidized cellulose/alginate loaded hydroxyapatite/graphene oxide beads in bone tissue engineering

**DOI:** 10.1186/s13065-025-01408-2

**Published:** 2025-02-26

**Authors:** Sawsan Dacrory, Lamiaa M. A. Ali, Safia Ouahrani-Bettache, Morgane Daurat, Mohamed El-Sakhawy, Peter Hesemann, Nadir Bettache, Samir Kamel

**Affiliations:** 1 Cellulose & Paper Department, National Research Centre, 33 El Bohouth St., Dokki, P.O. 12622, Giza, Egypt; 2 IBMM, Univ. Montpellier, CNRS, ENSCM, Montpellier, France; 3 IRIM, CNRS, University Montpellier, INSERM, Montpellier, France; 4 NanoMedSyn, Montpellier, France; 5 ChimEco CNRS UM 5021 CNRS-UM FR and Institut Charles Gerhardt de Montpellier, CNRS UMR 5253 CNRS-UM-ENSCM FR, Montpellier, France

**Keywords:** Sodium alginate, Tricarboxylic cellulose, Graphene oxide, Hydroxyapatite, Cytotoxicity

## Abstract

Bone regeneration is one of the most effective methods for treating bone defects. In this work, tricarboxylic cellulose/sodium alginate loaded with hydroxyapatite (HA) and/or graphene oxide (GO) was coagulated by calcium ions to create beads as scaffolds. In the first, cellulose was oxidized to water-soluble tricarboxylic cellulose (TCC) by 2,2,6,6‐tetramethylpiperidine-1-oxyl (TEMPO), periodate, and chlorite oxidation. HA was extracted from eggshells via microwave treatment, and GO was synthesized using the Hummer method. The structural behavior of the formed beads was meticulously investigated through various characterization techniques such as Fourier transform infrared spectroscopy (FT-IR), X-ray diffraction (XRD), thermogravimetric analysis (TGA), and scanning electron microscopy (SEM). The SEM images confirmed the formation of particles of micrometric size without any specific morphology. Incorporating GO or HA does not affect the morphologies of the materials on the micrometric scale. The cytocompatibility of different bead preparations was studied on murine mesenchymal stem cells. Moreover, the swellability in water and biodegradability by cellulase enzyme of prepared beads were studied. The results show that the prepared beads may be promising for bone tissue engineering.

## Introduction

The scarcity of existing tissues and organs and the growing need for bone engineering drove scientists to develop novel scaffolds [1, 2]. Consequently, due to their accessibility, cost, flexibility, and stability, synthetic scaffolds have emerged as an alluring replacement for natural graft materials [3]. Also, the optimal biodegradable scaffolds should immediately restore the mechanical integrity at the bone defect site and offer areas to direct the growth and repair of new bone tissue [4]. The selection of suitable materials for fabricating porous scaffolds is a crucial and often challenging aspect of bone tissue engineering, as it can significantly influence the success of the procedure. In the life sciences, tissue engineering and regenerative medicine are pioneer fields in replacing or restoring missing or damaged body parts [5]. Among these natural materials, hydroxyapatite (HA, Ca_10_(PO_4_)_6_(OH)_2_) is the major inorganic component of bones and teeth. Due to its biocompatibility and bioactivity, HA has been extensively used in various applications for bone repair [6]. Recently, natural materials such as fish bone, bovine bone, eggshells, etc., have been used to produce valuable biomaterials like HA. Eggshell is an abundant natural calcium resource with a % calcium carbonate content of 94% [7]. HA is similar to the mineral phase of natural bone, has been found to promote bone regeneration [8]. However, it has limitations-it cannot be fixed on the defect's position and cannot accommodate its uneven shape, which restricts its application in bone repair [9]. To overcome this, it has been combined with polymers, a significant innovation, to fabricate scaffolds or pastes [10]. Different biopolymers, including cellulose [11], alginate [12], catechol [13], and hyaluronic acid, have been used to fabricate different biomimetic mineralization systems with biocompatibility and high colloidal stability. Cellulose and alginate are renewable and abundant natural biopolymers that are biodegradable, biocompatible, cheap, and non-toxic. The most abundant natural biopolymer is cellulose, which consists of anhydro-d-glucopyranose units with three hydroxyl groups *per* unit. Oxidation is one of the most common chemical derivatization of cellulose. It allows the conversion of hydroxyl groups of cellulose to carboxylic acid or carboxylate groups [14, 15]. The formation of carboxyl groups at the C2, C3, and C6 leads to water-soluble or swellable oxidized cellulose, a key step in the creation of various cellulose-derived composites with practical applications. For example, 3D scaffold composites were prepared by coating HA with carboxymethyl cellulose, a material with high biodegradability and the ability to form an appetite layer on the surface [1]. Also, a porous collagen-carboxymethyl cellulose/HA composite was fabricated for biomimetic mineralization, demonstrating the potential of these composites in tissue engineering. The composite, with its 3D porous structure, showcased a compressive strength that was dependent on the collagen-to-carboxymethyl cellulose ratio. The composite was biodegraded in 8 weeks and exhibited a high relative growth rate of wild-type mouse embryonic fibroblast cells [16].

The other abundant natural biopolymer is sodium alginate (SA). SA can be extracted from brown algae. It is a linear copolymer of the β-1,4-glycosidic linked α-l-mannuronic acid and has widespread applications for tissue engineering, drug delivery, and biological studies [2]. SA is rich in OH and COOH groups; thus, it is readily cross-linked by various polyvalent ions, such as Ca^2+^, which modulate the synthesis of HA [17]. The complexation of Ca^2+^ with alginate controls the release of Ca^2+^, suppressing the growth of HA crystals, leading to the hybridization of alginate into HA crystals [18]. In addition to the above biopolymers, graphene oxide (GO) has unique characteristics that have attracted great attention. GO, a single layer of sp^2^-hybridized carbon atoms with hydroxyl, epoxy, and carboxyl groups distributed on its surface, is produced from multilayers graphite by different methods [19–21]. Its unique characteristics, such as mechanical strength and stiffness, superior electrical conductivity, and surface functionality, make GO a fascinating material for tissue engineering [22]. Several studies have demonstrated the biocompatibility of graphene in vitro and in vivo [23].

Based on the literature review, the presence of GO in the scaffold supports osteoblast adhesion [24]. Additional HA is the primary ingredient in bone; it is regarded as a suitable scaffold material for bone tissue creation. HA is an artificial synthetic with low immunological rejection, great biosecurity, and bioactivity. It offers chemical stability and the ability to conduct bone, creating a condition supporting the differentiation of seed cells into osteoblasts. Furthermore, HA contains calcium and phosphorus to aid the body's metabolism [25].

In this work, we have developed a new scaffold in the form of beads for potential application in enhancing bone regeneration. This scaffold uses SA, TCC, GO, and HA as recycled eggshells with CaCl_2_ as a cross-linker and has undergone a comprehensive characterization process using FTIR, XRD, SEM, and TGA techniques. Additionally, its cytotoxicity was evaluated in vitro on cell culture to ensure the reliability of our findings.

## Material and methods

### Materials

Cellulose pulp with 96% α-cellulose was supplied from Qena Company, Egypt. Eggshells were collected. Sodium alginate (SA) and Graphite (G) powder (99.9%) were provided by Fisher Scientific UK. Potassium permanganate (> 99%) and hydrogen peroxide (30%) were purchased from Bio Basic Canada Inc. and Carl Roth GmbH, respectively. Sodium nitrate (99.99%) was supplied by Sd Fine-CHEM Limited (India). Sodium metaperiodate (NaIO_4_), NaBr, and 2,2,6,6 tetramethylpiperidine-1-oxyl (TEMPO) were purchased from Sigma Aldrich. All chemicals and reagents used were in analytical grade and were used as received. The 3-(4,5-dimethylthiazol-2-yl)-2,5-diphenyltetrazolium bromide (MTT) was purchased from Sigma-Aldrich, Saint-Quentin-Fallavier, France. Cellulase enzyme (from *Aspergillus niger*) was purchased from Sigma (C-1184). Additional cellulase source was obtained from the local fungal strain MHN-EGY [26].

### Methods

#### Preparation of tricarboxylic cellulose (TCC)

The preparation of tricarboxylic cellulose involved a meticulous sequence of three steps: TEMPO, periodate, and chlorite oxidations.

The first step involved a thorough procedure. Cellulose (5 g) was mixed with sodium bromide (0.8 g, 8 mmol) and TEMPO (0.08 g, 0.5 mmol) in 500 mL of distilled water after adding sodium hypochlorite solution (50 mL, 10%) and pH adjustment to 10, at the end of the process the pH adjustment to 7 and centrifugation at 7000 rpm. The product was refined through a series of water additions, dispersions, and centrifugations, followed by a week-long dialysis against deionized water for thorough cleaning.

In the second step, 46 mmol of sodium metaperiodate was added to TEMPO-oxidized cellulose diluted to 1% in distilled water and heated to 60 °C in a water bath. The reaction container was then covered with aluminum foil to prevent the photo-induced breakdown of the periodate. After 3 h, the dialdehyde oxidized cellulose was filtered and rinsed with distilled water.

Finally, in the third step, 60 mL of acetic acid (20%) was gradually added to sodium chlorite (50 mmol/40 mL H_2_O_2_); acetic acid was progressively added to create a yellowish color. This mixture was added to dialdehyde oxidized cellulose, 4.5 g of, with a consistency of 4.5%, and stirred for 48 h at room temperature. TCC was filtered and cleaned with deionized water. The yield of the prepared TCC was between 80 and 85% [27].

#### Preparation of graphene oxide (GO)

The GO nanosheet has been prepared through the modified Hummer method, as described in our previous work [28]. Briefly, 0.225 g of graphite powder was added to a mixture of sulfuric acid (27 mL) and phosphoric acid (3 mL). To this mixture, potassium permanganate (1.32 g) was added slowly and stirred for 6 h until the solution became dark green. Next, hydrogen peroxide (0.675 mL) was dropped slowly and stirred for 10 min to remove the excess permanganate. Finally, hydrochloric acid (10 mL) and deionized water (30 mL) were added. Then, the supernatant was separated, and the solid was washed with deionized water 3 times. Finally, a black product was obtained after drying at 90 °C for 24 h.

#### Preparation of Hydroxyapatite (HA) from Eggshells

The collected eggshells underwent a rigorous cleaning process, being immersed in boiling water to remove all surface contaminants and the inner membrane. The dried eggshells were then ground into powder in a mortar and subjected to a thorough immersion in sodium hypochlorite to remove all organic components. The resulting solid was washed repeatedly with water and dried in a vacuum oven at 110 °C. To obtain HA, 1 g of eggshell powder was mixed with EDTA solution (0.1 M) to form a Ca–EDTA complex. Next, 0.06 M of Na_2_HPO_4_ solution was slowly added, pH was adjusted to 13 by sodium hydroxide solution and stirred for 30 min, followed by microwave irradiation for 10 min. The HA was obtained as a white solid after drying at 110 °C [29].

#### Preparation of bead scaffolds

A mixture of aqueous SA solution (50 mL, 2%) and TCC (0.5 g) was prepared with a magnetic stirrer. GO and/or HA were ultrasonicated for 10 min to load HA and GO onto alginate beads. This mixture was added to the SA/TCC solution under magnetic stirring. The whole mixture was then added drop-wise using a peristaltic pump at a 6 mL/min flow rate to a 10% CaCl_2_ solution under constant and gentle magnetic stirring. The solution was stirred to guarantee solution density uniformity and prevent aggregation. Afterward, the beads were kept in a CaCl_2_ solution for 2–3 days without stirring, then washed with deionized water three to four times and stored in a refrigerator [17]. We conducted a thorough investigation of four different compositions, with the exact quantities of the components summarized in Table 1.

**Table 1:** The composition of the prepared beads

Code	SA (g)	TCC (g)	HA (g)	GO (g)
B1	1	0.5	00	00
B2	1	0.5	0.1	00
B3	1	0.5	00	0.1
B4	1	0.5	0.05	0.05

### Characterizations

#### Fourier-transform infrared (FT-IR) spectroscopy

FT-IR was recorded in the 400–4000 cm^−1^ range on the (Shimadzu 8400S) FT-IR Spectrophotometer range using the KBr disk method.

#### The X-ray diffraction (XRD)

XRD patterns were investigated on a Diano X-ray diffractometer using a CuKα radiation source energized at 45 kV and a Philips X-ray diffractometer (PW 1930 generator, PW 1820 goniometer) with CuK radiation source (λ = 0.15418 nm), at a diffraction angle range of 2θ from 5 to 70° in reflection mode.

#### Thermogravimetric analysis (TGA)

The thermal stability of beads was carried out using a thermogravimetric analysis (TGA) Perkin-Elmer (STA6000), with a heating rate (10 °C/min). The temperature ranged from room temperature up to 900 °C under air atmosphere.

#### Scanning electron microscopy (SCM)

The samples’ morphology was performed by using a Hitachi S4800 Scanning electron microscope at an acceleration voltage of 120 kV.

#### Swelling degree

The swelling of the prepared beads was monitored gravimetrically. A dry piece of bead was immersed in distilled water and allowed to swell for precisely 1 day at room temperature. Afterward, the beads were removed from the water; the excess water was removed using filter paper and weighed [28]. The swelling ratio (*Q*) was expressed as the percentage of weight gain compared to the dry weight as follows:

$$Q\% = \frac{{{\text{mt }}{-}{\text{ m}}0}}{{{\text{m}}0}} \times 100$$

where m_t_ and m_o_ are the wet and dry weights of the beads, respectively. *Q*, the swelling ratio, is a key metric in our study, calculated as the grams of water per gram of bead sample. Three parallel experiments were carried out on every bead, and the main values were taken.

#### Cellulase assay

The suitability of beads for degradation by cellulase enzyme has been determined by measuring the cellulase enzyme activity [30]. 1 mL of diluted enzyme solution was added to 1 mL of citrate buffer (0.05 mM, pH 4.8) containing 50 mg of bead, and the mixture was incubated for 1 h at 50 °C. At the same time, a standard curve of glucose was constructed. Reducing sugars were determined as glucose, and one cellulase unit of enzyme is defined as the enzyme amount that liberates 1 micromole of reducing sugars, expressed as glucose, per minute, under specified conditions [26].

#### Cytotoxicity

Murine mesenchymal stem cells were gently given by Dr. Muriel Amblard, IBMM, France. Cells were maintained in Dulbecco’s Modified Eagle Medium (DMEM) supplemented with 10% fetal bovine serum and 1% penicillin/streptomycin at 37 °C and 5% CO_2_. For the cytotoxicity test, cells were seeded in 96 well plates at a density of 5000 cells per well. Twenty-four hours after seeding, cells were treated with different bead preparations at concentrations ranging from 0 to 400 µg/mL. Cells were incubated with beads for 3 days, and then the cell viability was assessed using an MTT assay. Briefly, cells were incubated with MTT at a final concentration of 0.5 mg/mL for 4 h. The supernatant was aspirated, and the formed violet precipitated crystals were dissolved with ethanol/DMSO solution (1:1, v/v), followed by shaking for 20 min. Absorbance was read at 540 nm using a Multiskan SkyHigh Microplate Spectrophotometer (Fisher Scientific SAS, France). The percentages of viable cells were calculated using the following equation:

$${\text{Viable}}\;{\text{cells }}\left( \% \right) = \frac{{{\text{Ab}}_{{{\text{test}}}} }}{{{\text{Ab}}_{{{\text{control}}}} }} \times 100$$

The experiment was repeated three times.

## Result and discussion

### Synthesis and characterization of the components

The preparation process of biocompatible bead scaffolds involves three starting materials:

- TCC, obtained from cellulose via oxidation.
- GO, prepared from graphite via oxidation.
- HA, obtained from eggshells.

**Scheme 1:**
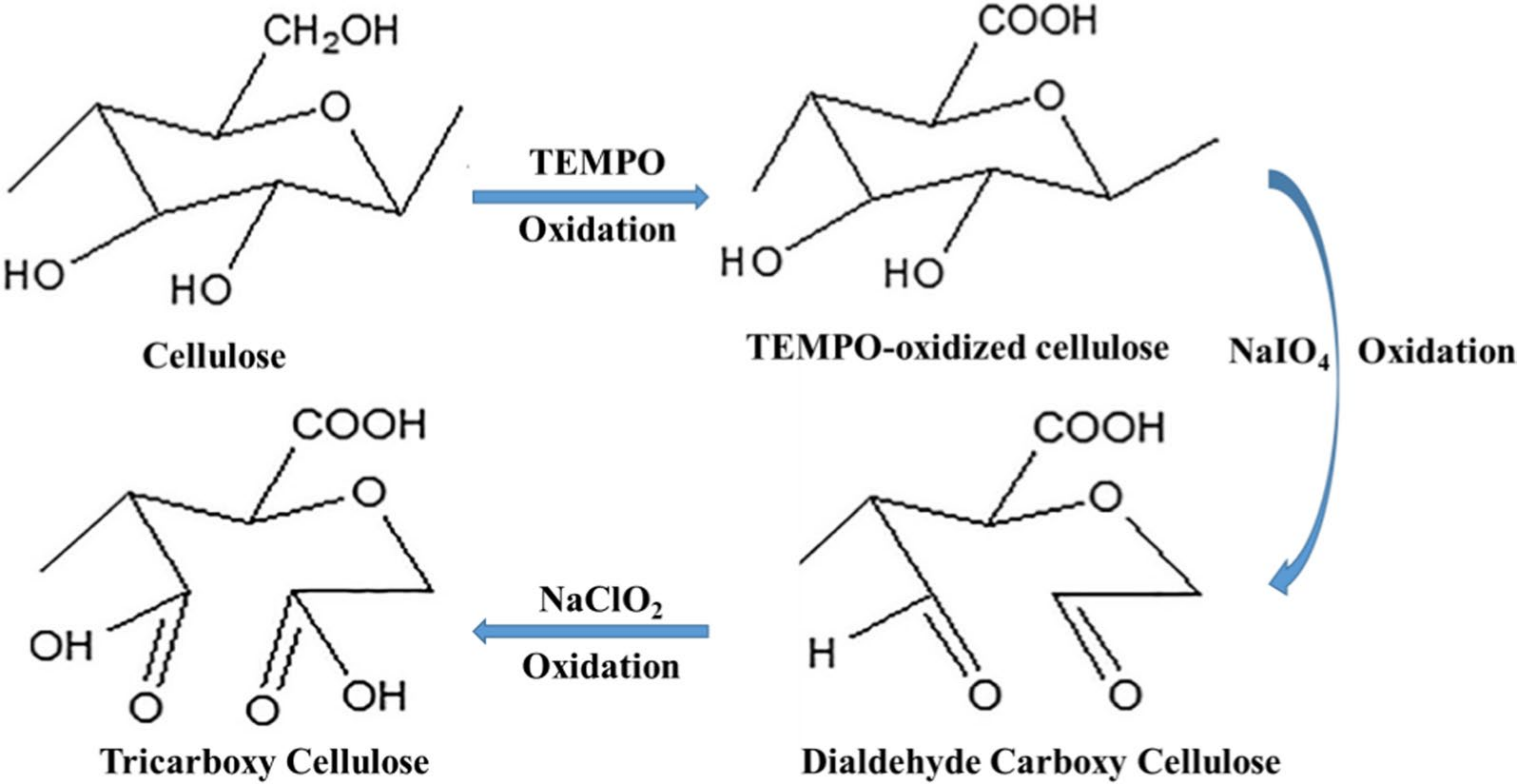
Cellulose oxidation onto tricarboxy cellulose (TCC)

**Fig. 1:**
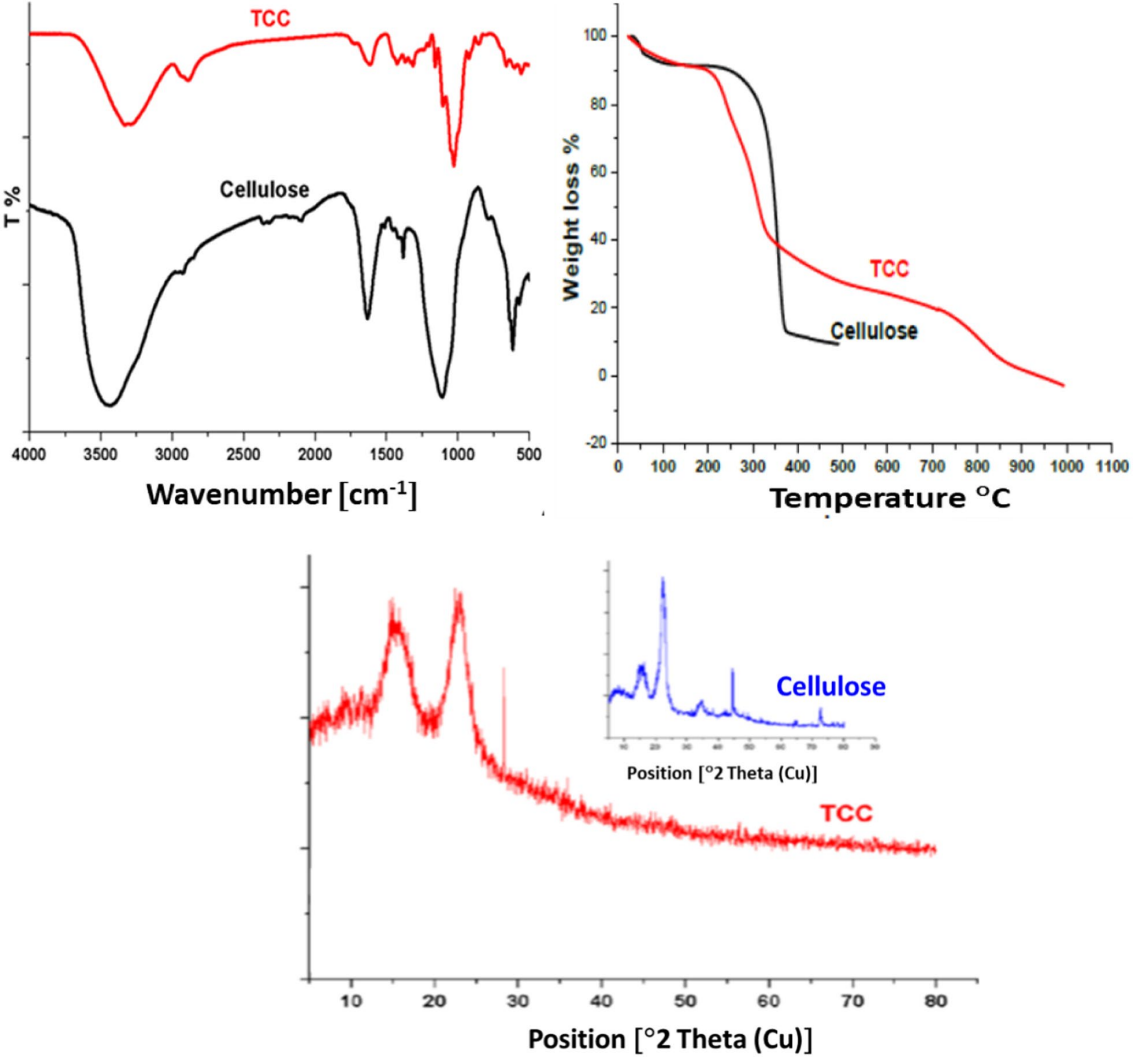
FTIR-Spectra, XRD, and TGA of cellulose and TCC

Cellulose was oxidized to form carboxyl groups at the glucosyl units C2, C3, and C6. First, as a selective oxidation agent, TEMPO-mediated oxidation converted cellulose’s primary hydroxyl group at C6 to carboxylates, followed by selective periodate oxidation of hydroxyl groups at C2 and C3 onto aldehyde groups. Finally, the dialdehyde groups at C2 and C3 were oxidized to dicarboxylic acids by hypochlorite oxidation (Scheme 1) [31]. The oxidation process was followed via FT-IR-spectroscopy, XRD, and TGA of the starting material, intermediates, and product. From the FT-IR spectra (Fig. 1), the intensity of a broad peak at around 3400 cm^−1^, which is attributed to the stretching vibration of OH groups, was decreased by oxidation, while the peak at 2900 cm^−1^ revered to sp3 hybridized C–H stretching was increased. A new weak peak at 1740 cm^−1^ was observed after oxidation, attributed to carbonyl groups in the free COOH group [32]. Also, to confirm the oxidation of cellulose, X-ray diffractograms of cellulose and oxidized cellulose were recorded (Fig. 1). The X-ray diffraction patterns show two diffraction rays at 2θ = 16.5° and 22°. The intensity of the ‘amorphous’ peak decreased due to a loss of amorphous components during the oxidation reaction [32]. The comparison of the thermogravimetric analysis of oxidized with native cellulose (Fig. 1) indicated that the oxidation process considerably impacted their thermal stability. The degradation of TCC started at a significantly lower temperature (200 °C instead of 350 °C for pristine cellulose). A similar trend was reported by Fukuzumi et al. and was explained by the formation of carboxylate groups on the surface of cellulose and in disordered regions [33].

**Fig. 2:**
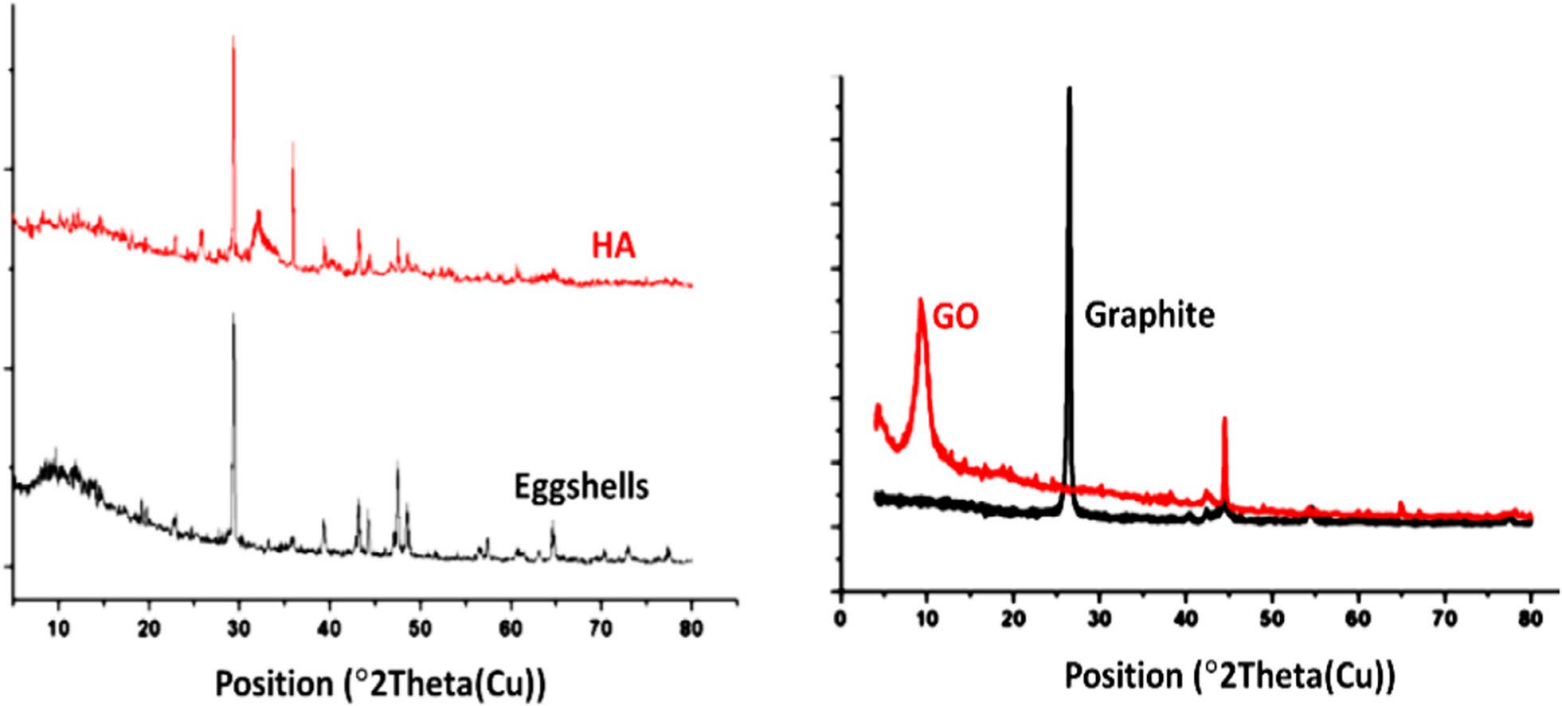
XRD of Eggshell, HA, Graphite, and GO

GO was prepared from graphite using a modified Hummer’s oxidation method. The oxidation process was monitored by X-ray diffraction. Figure 2 depicts the XRD patterns of pristine graphite and GO. At the same time, the diffractogram of graphite exhibits a sharp peak at 2q ~ 27°; an intense diffraction ray can be seen in the case of GO at 2θ ~ 10°.

The complete disappearance of the diffraction ray at 2q ~ 27° confirms the introduction of oxygen-containing functionalities such as hydroxyl, epoxy, and carboxyl groups, as reported by Ghanem et al. [34].

HA was prepared by recycling eggshells via microwave treatment. After the removal of the organic pollutants from eggshells, the dried powder was dissolved in an EDTA solution according to the following equation:
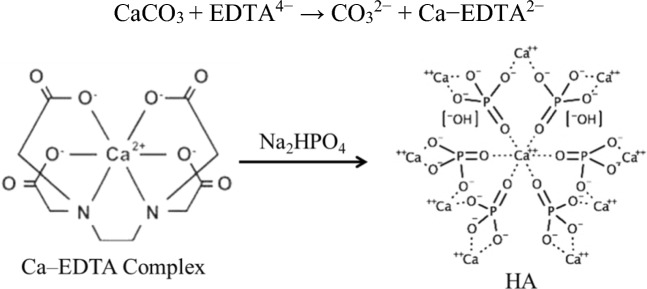


A clear solution was formed by adding sodium phosphate due to the stability of the Ca–EDTA complex in basic media. Under microwave irradiation, the Ca–EDTA complex decomposes, and HA crystals with particular architectures are formed [35]. Figure 2 shows the XRD pattern of eggshell powder and HA. It can be noted that the amorphous halo region at 10° < 2θ < 20° in eggshells pattern also appears in the diffractograms of HA. At the same time, new peaks appeared at 2θ = 35 and 37°, indicating the presence of Ca(H_2_PO_4_)_2_, as these diffraction rays match with the standard HA (JCPDS file number 9–0432 [36].

### Preparation and characterization of bead scaffolds

**Fig. 3:**
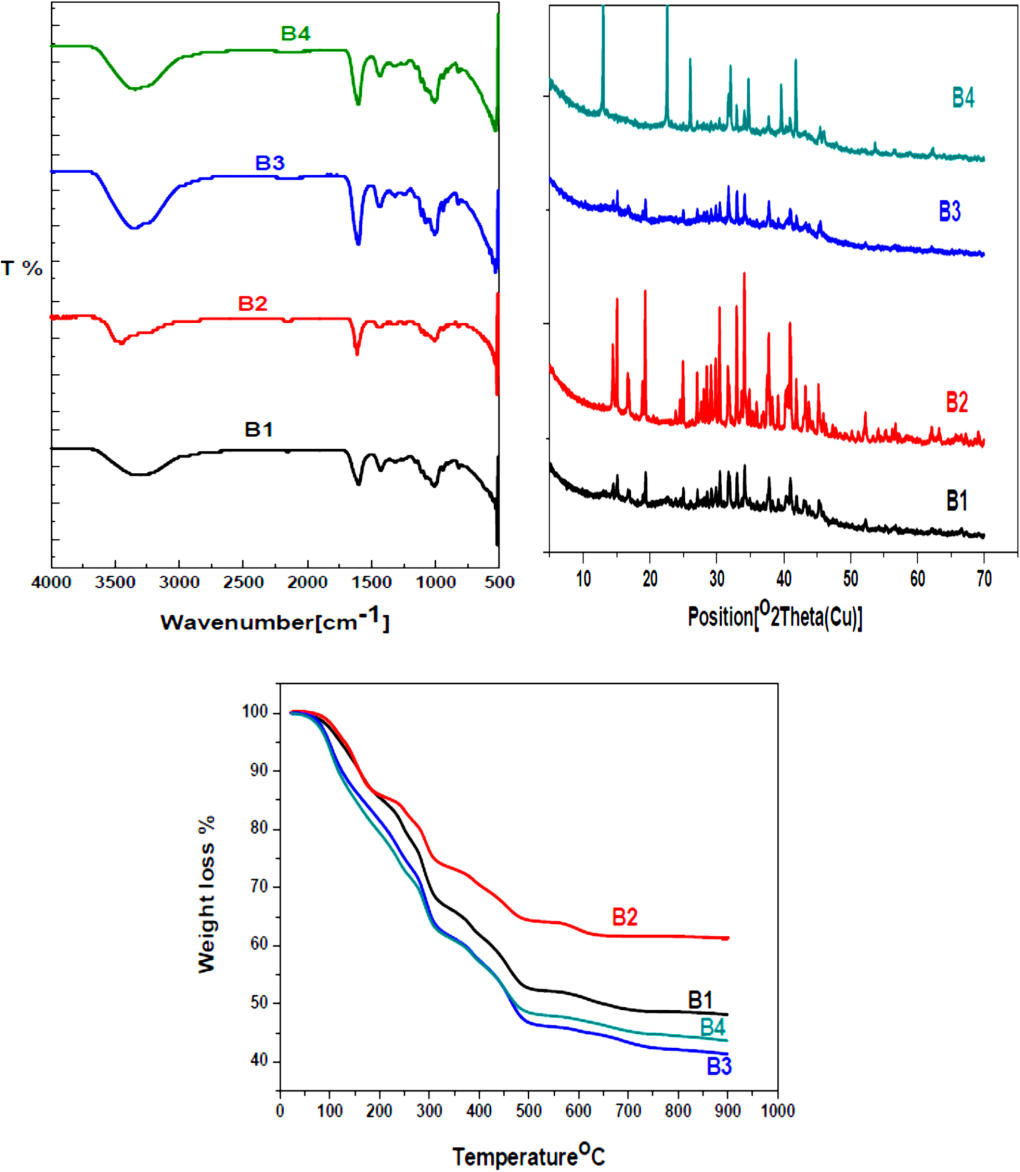
FT-IR-Spectra, XRD, and TGA of the bead scaffolds

GO and/or HA-reinforced SA/TCC bead scaffolds were prepared using Ca^2+^ as a coagulation agent. The FT-IR spectra, X-ray diffractograms, and TGA thermograms of the scaffolds are shown in Fig. 3. First, the scaffolds were characterized by FT-IR spectroscopy (Fig. 3). The FT-IR spectrum of SA shows intense absorption bands at 3244, 2920, 1591, and 1024 cm^−1^ that can be assigned to stretching vibrations of O–H bonds, stretching vibrations of aliphatic C–H, asymmetric stretching vibrations of –COO–, and elongation of C–O groups, respectively [37]. By mixing SA with TCC, the –OH and –COO– bands (~ 3244 and 1591 cm^−1^) are weakened, and their broadening suggests the formation of strong hydrogen bonding between these two components (B1). A further intensity decrease and peak broadening appeared after the addition of HA due to the complex formation of HA with SA and TCC (B2) [38].

The XRD patterns of SA and bead scaffolds, as depicted in Fig. 3. SA, typically semi-crystalline or crystalline, owes its structure to the strong intermolecular hydrogen bonding between chains. The distinct diffraction peaks at 2θ = 13.5°, 22°, and 39°, corresponding to (110), (200), and others from polyguluronate, polymannuronate, and amorphous halo [39]. The XRD patterns of B2 and B4 show relatively strong peaks at 2θ ~ 25.9°, 31.8°, 39.8°, 46.7°, and 48.9°, corresponding to the planes of (002), (211), (310), (222), and (213) of HA, respectively, confirming the crystalline nature of the HA components in these beads (B2 and B4) [40]. The absence of complete peaks in B3 suggests that the GO is dispersed at the molecular level in the SA and TCC, playing a crucial role in their composition.

The TGA characterized the thermal stability of bead scaffolds. Figure 3 presents the TGA result of B1-4 tested in an N_2_ protective atmosphere. When it is below 180 °C, the weight loss is mainly due to the moisture evaporation in beads. The second stage was 180–300 °C, corresponding to the decomposition of labile oxygen-containing functional groups [41]. In the case of B2, the decomposition shifted to a higher temperature than B1; this result is probably due to the loading of HA leading to the thermal stability. The loading of GO enhanced the thermal degradation due to the decomposition of the carboxyl group. Above 500 °C, B1, B3, and B4 further reduced weight due to the impressive contribution of bulk pyrolysis to the carbon skeleton [42].

### Surface morphology by SEM

**Fig. 4:**
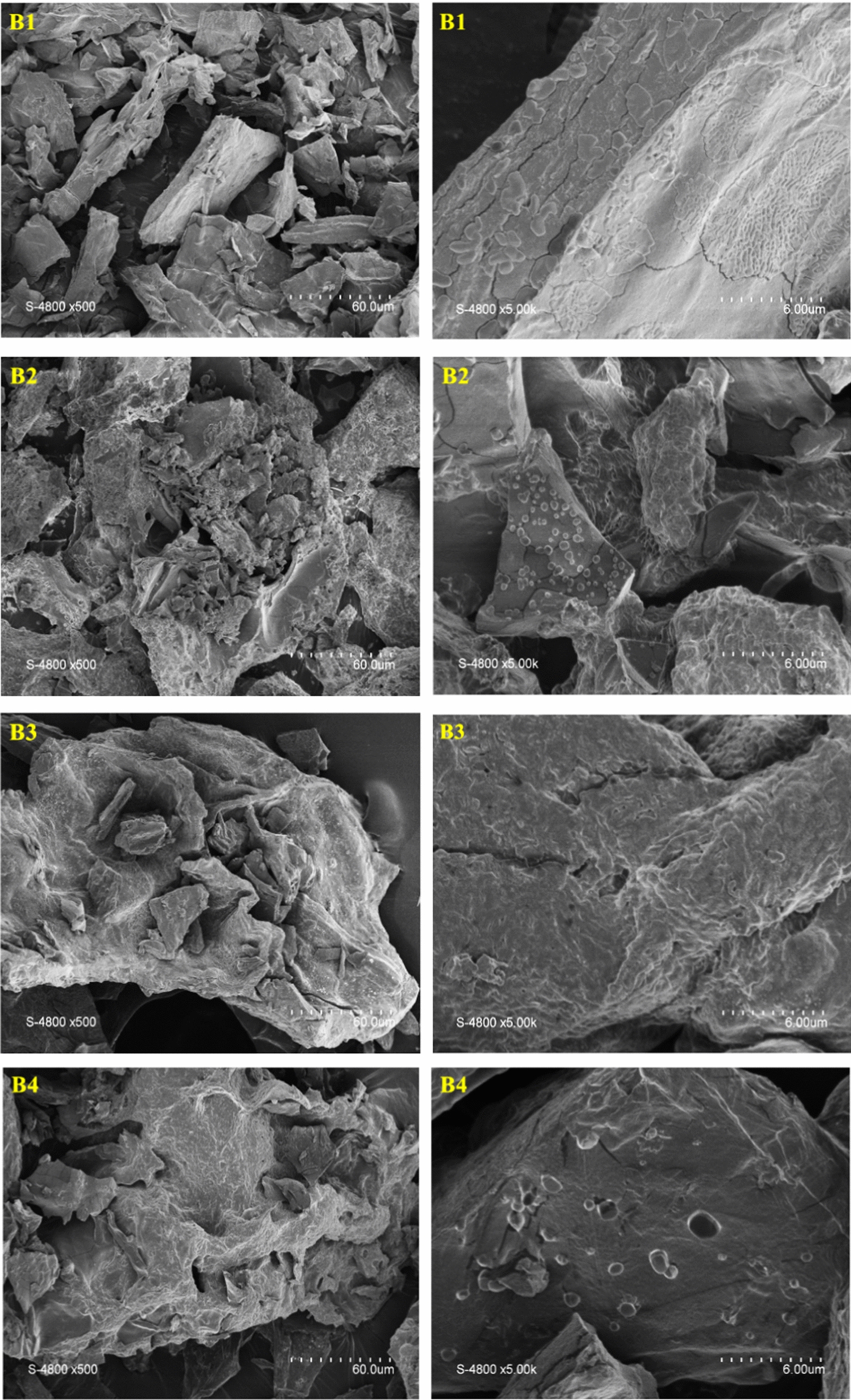
SEM images of the beads (B1–B4)

Figure 4 shows the SEM images of the four beads with different magnifications (× 500/× 5000). All beads B1–B4 consist of dense particles of micrometric size without specific morphologies. Adding HA and/or GO does not strongly impact the formed beads’ morphologies.

### Swelling behavior

**Fig. 5:**
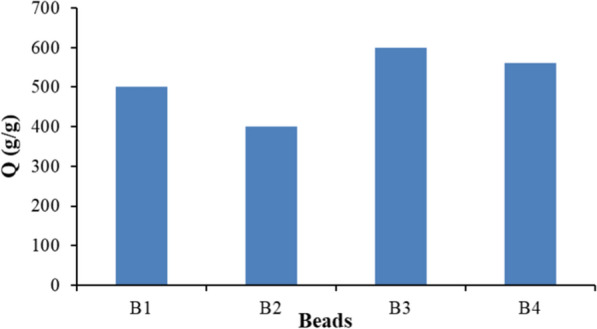
Swelling behavior of beads

TCC was mixed with SA solution, then crosslinked by Ca^2+^ to obtain the beads hydrogels with HA and/or GO for scaffolding. A key sign of a scaffold’s potential for therapeutic use is how effectively it swells. Appropriate swellability increases the scaffold's surface area and encourages nutrient transfer, encouraging more cell attachment and penetration. The swelling of hydrogels is attributed mainly to electrostatic repulsion between the charged polymer chains and their hydrophilicity [43]. This study studied the swelling behavior as the swelling ratio of beads in distilled water with pH 7.1 (Fig. 5). B1 showed the lowest swellability, which can be explained by the fact that the TCC molecular chain contained many –OH and –COOH groups, which could form intermolecular hydrogen bonds with SA. Its tight fiber network structure and good barrier properties could also limit the swelling. Introducing HA into the beads decreased the swelling ratio as HA could contract and restrict the movability of the TCC and SA chains, diminishing the swelling ratio [44].

On the other hand, incorporating GO into beads increased the swelling ratio. This is due to water interacting with the –OH functional groups. Since GO has several –OH and other hydrophilic functional groups, these functional groups tend to interact with water due to enhanced hydrogen bonding. More hydrogel swelling results from excessive hydrogen bonding [45].

### Degradability of beads

The susceptibility of beads to be degraded was assessed by studying their ability to be degraded by commercial cellulase enzymes such as Sigma and from local fungal strains. Cellulase enzyme is classified as an exoglucanase and cleaves β-1,4-glycosidic linkages of cellulose chains to enable further degradation of the chains [46]. The enzyme activity is affected by the structure of both substrate and enzyme. Table 2 showed that the tested beads degraded at different rates and depended on the beads’ composition, so the biodegradation occurred differently. Beads B1, B2, and B4 degraded better than other beads. This result may be because hybrid materials conferred some chemical stability to the final product.

**Table 2:** Effect of cellulases against different beads

Bead	Sigma cellulase activity (U/mL)	% to control	Local fungal cellulase activity (U/mL)	% to control
TCC (control)	1.59	100	0.79	100
B1	1.13	71.07	0.72	91.14
B2	1.18	74.21	0.68	86.08
B3	1.04	65.41	0.57	72.15
B4	1.22	76.73	0.75	94.94

### Cytotoxicity study

The biological responses of a composite containing GO and HA depend on the availability of functional groups for the apatite nucleation. The literature reports that a biomaterial’s crystallinity degree and dissolution rate can influence its in vitro and in vivo bioactivity properties [47, 48]. Some authors have evaluated the cellular metabolic activity of HA and HA-GO composites, and the cytotoxicity of these composites indicated a remarkable 95% cell viability when compared to the positive control (cells only) [49]. In another study, MTT results demonstrated an adequate growth of pre-osteoblastic MC3T3-E1 cells on HA-GO composites due to a favorable surface for cell adhesion and proliferation [50]. Therefore, the chemical presence of GO indicated better biological results. The presence of OH groups on the surface of GO sheets has a synergistic effect with ion exchange from Ca, which speeds up the kinetics of the biomineralization process [51].

**Fig. 6:**
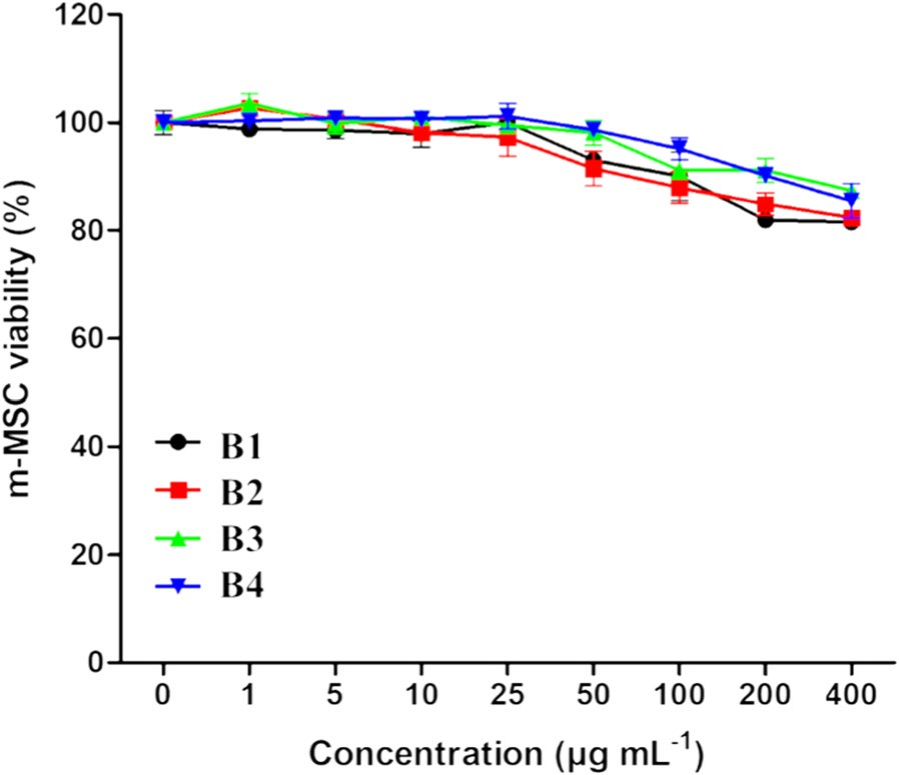
Cytotoxicity study of beads on murine mesenchymal stem cells treated with different concentrations of beads for 72 h. Results are presented as mean ± SEM (n = 3)

In the present work, the cytotoxicity of the prepared beads was investigated on murine mesenchymal stem cells (m-MSC) (Fig. 6). After 3 days of incubation with varying concentrations of bead preparations, a similar cytotoxic response was observed. Notably, all beads’ cell viability was above 80%, even at the highest concentration of 400 µg/mL. At this concentration, cell viability values were 81.5 ± 1.9%, 82.4 ± 2.4%, 87.4 ± 1.8% and 85.5 ± 5.6% for B1, B2, B3 and B4, respectively. Suggesting these samples as a promising material for bone tissue regeneration.

## Conclusion

In this study, eggshell waste has been successfully recycled to HA using a simple and eco-friendly method with the assistance of microwave irradiation. Bead scaffolds were prepared by crosslinking SA and TCC using Ca^2+^ and loading HA and/or GO. FT-IR, XRD, and TGA analysis confirmed all bead structures. The effect of HA and GO on the swellability and biodegradability were investigated. Importantly, the cell viability assay revealed the low cytotoxic effect of all prepared beads on murine mesenchymal stem cells after 3 days of incubation with 400 µg/mL concentration concentrations**,** underscoring the safety of these preparations for potential use in tissue engineering applications.

## Data Availability

All data generated or analysed during this study are included in this published article.

## Abbreviations

HA: Hydroxyapatite
G: Graphite
GO: Graphene oxide
TCC: Tricarboxylic cellulose
TEMPO: 2,2,6,6‐Tetramethylpiperidine-1-oxyl
SA: Sodium alginate

## References

[1] Sayed M, et al. 3D carboxymethyl cellulose/hydroxyapatite (CMC/HA) scaffold composites based on recycled eggshell. J Appl Pharm Sci. 2018;8(3):023–30.

[2] Lee KY, Mooney DJ. Alginate: properties and biomedical applications. Prog Polym Sci. 2012;37(1):106–26. 22125349 10.1016/j.progpolymsci.2011.06.003 PMC3223967

[3] Damien CJ, Parsons JR. Bone graft and bone graft substitutes: a review of current technology and applications. J Appl Biomater. 1991;2(3):187–208. 10149083 10.1002/jab.770020307

[4] Kim B-S, Mooney DJ. Development of biocompatible synthetic extracellular matrices for tissue engineering. Trends Biotechnol. 1998;16(5):224–30. 9621462 10.1016/s0167-7799(98)01191-3

[5] Maleki M, et al. Graphene oxide: a promising material for regenerative medicine and tissue engineering. Biomol Concepts. 2020;11(1):182–200. 34233430 10.1515/bmc-2020-0017

[6] Chen Y, et al. Potential of a sensitive uric acid biosensor fabricated using hydroxyapatite nanowire/reduced graphene oxide/gold nanoparticle. Microsc Res Tech. 2020;83(3):268–75. 31729094 10.1002/jemt.23410

[7] Andronescu E, et al. Synthesis and characterization of collagen/hydroxyapatite: magnetite composite material for bone cancer treatment. J Mater Sci Mater Med. 2010;21(7):2237–42. 20372983 10.1007/s10856-010-4076-7

[8] Su W, et al. RhBMP-2 and concomitant rapid material degradation synergistically promote bone repair and regeneration with collagen–hydroxyapatite nanocomposites. J Mater Chem B. 2018;6(26):4338–50. 32254509 10.1039/c8tb00405f

[9] Farokhi M, et al. Silk fibroin/hydroxyapatite composites for bone tissue engineering. Biotechnol Adv. 2018;36(1):68–91. 28993220 10.1016/j.biotechadv.2017.10.001

[10] Ni P, et al. Electrospun preparation and biological properties in vitro of polyvinyl alcohol/sodium alginate/nano-hydroxyapatite composite fiber membrane. Colloids Surf, B. 2019;173:171–7. 10.1016/j.colsurfb.2018.09.074 30292025

[11] Sadh PK, Duhan S, Duhan JS. Agro-industrial wastes and their utilization using solid state fermentation: a review. Bioresour Bioprocess. 2018;5(1):1–15.

[12] Ma X, et al. Biologically inspired, catechol-coordinated, hierarchical organization of raspberry-like calcium phosphate nanospheres with high specific surface area. J Mater Chem B. 2018;6(22):3811–9. 32254843 10.1039/c7tb03156d

[13] Chen Z, et al. Controlled mineralization by extracellular matrix: monodisperse, colloidally stable calcium phosphate-hyaluronan hybrid nanospheres. Chem Commun. 2010;46(8):1278–80. 10.1039/b918835e 20449276

[14] Baseer RA, et al. A biodegradable film based on cellulose and thiazolidine bearing UV shielding property. Sci Rep. 2022;12(1):1–15. 35550531 10.1038/s41598-022-11457-5 PMC9098501

[15] Al Kiey SA, Hasanin MS, Dacrory S. Potential anticorrosive performance of green and sustainable inhibitor based on cellulose derivatives for carbon steel. J Mol Liq. 2021;338: 116604.

[16] He X, et al. A porous collagen-carboxymethyl cellulose/hydroxyapatite composite for bone tissue engineering by bi-molecular template method. Int J Biol Macromol. 2019;137:45–53. 31220495 10.1016/j.ijbiomac.2019.06.098

[17] Fei Y, et al. Adsorptive removal of ciprofloxacin by sodium alginate/graphene oxide composite beads from aqueous solution. J Colloid Interface Sci. 2016;484:196–204. 27614043 10.1016/j.jcis.2016.08.068

[18] Ma L, et al. Synthesis and characterization of injectable self-healing hydrogels based on oxidized alginate-hybrid-hydroxyapatite nanoparticles and carboxymethyl chitosan. Int J Biol Macromol. 2020;165:1164–74. 33038398 10.1016/j.ijbiomac.2020.10.004

[19] Dacrory S. Development of mesoporous foam based on dicarboxylic cellulose and graphene oxide for potential oil/water separation. Polym Bull. 2021;79:1–12.

[20] Neelgund GM, Oki A, Luo Z. In situ deposition of hydroxyapatite on graphene nanosheets. Mater Res Bull. 2013;48(2):175–9. 25110359 10.1016/j.materresbull.2012.08.077 PMC4124456

[21] Kamel S, et al. Graphene’s structure, synthesis, and characterization; a brief review. Egypt J Chem. 2019;62(Special Issue (Part 2) Innovation in Chemistry):593–608.

[22] Shang L, et al. Graphene and graphene oxide for tissue engineering and regeneration. In: Theranostic bionanomaterials. Elsevier; 2019. p. 165–85.

[23] Alaghmandfard A, et al. Recent advances in the modification of carbon-based quantum dots for biomedical applications. Mater Sci Eng, C. 2021;120: 111756. 10.1016/j.msec.2020.111756 33545897

[24] Devi GVY, et al. Fucoidan-incorporated composite scaffold stimulates osteogenic differentiation of mesenchymal stem cells for bone tissue engineering. Mar Drugs. 2022;20(10):589. 36286414 10.3390/md20100589 PMC9604642

[25] Qi J, et al. Current biomaterial-based bone tissue engineering and translational medicine. Int J Mol Sci. 2021;22(19):10233. 34638571 10.3390/ijms221910233 PMC8508818

[26] Hussien AH, et al. Promising biodegradable composite derived from corn straw fiber and waste Polyethylene. Egypt J Chem. 2021;64(6):3205–13.

[27] Abou-Zeid RE, et al. Novel method of preparation of tricarboxylic cellulose nanofiber for efficient removal of heavy metal ions from aqueous solution. Int J Biol Macromol. 2018;119:207–14. 30036619 10.1016/j.ijbiomac.2018.07.127

[28] Abdelaziz AM, et al. Protective role of zinc oxide nanoparticles based hydrogel against wilt disease of pepper plant. Biocatal Agric Biotechnol. 2021;35: 102083.

[29] Kumar GS, Thamizhavel A, Girija E. Microwave conversion of eggshells into flower-like hydroxyapatite nanostructure for biomedical applications. Mater Lett. 2012;76:198–200.

[30] Mandels M, Hontz L, Nystrom J. Enzymatic hydrolysis of waste cellulose. Biotechnol Bioeng. 1974;16(11):1471–93. 10.1002/bit.22603 19937801

[31] Kim U-J, et al. Periodate oxidation of crystalline cellulose. Biomacromol. 2000;1(3):488–92. 10.1021/bm0000337 11710141

[32] Dacrory S, Kamel S. Magnetic composite based on cellulose and GO for latent fingerprint visualization. Egypt J Chem. 2022;65(7):1–6.

[33] Fukuzumi H, et al. Transparent and high gas barrier films of cellulose nanofibers prepared by TEMPO-mediated oxidation. Biomacromol. 2009;10(1):162–5. 10.1021/bm801065u 19055320

[34] Ghanem AF, et al. Synergistic effect of zinc oxide nanorods on the photocatalytic performance and the biological activity of graphene nano sheets. Heliyon. 2020;6(2): e03283. 32055730 10.1016/j.heliyon.2020.e03283 PMC7005451

[35] Liu J, et al. Rapid formation of hydroxyapatite nanostructures by microwave irradiation. Chem Phys Lett. 2004;396(4–6):429–32.

[36] Bouropoulos N, Stampolakis A, Mouzakis DE. Dynamic mechanical properties of calcium alginate-hydroxyapatite nanocomposite hydrogels. Sci Adv Mater. 2010;2(2):239–42.

[37] Mekheimer RA, et al. Green, three component highly efficient synthesis of 2-amino-5, 6, 7, 8-tetrahydro-4-H-chromen-3-carbonitriles in water at ambient temperature. Green Chem Lett Rev. 2010;3(3):161–3.

[38] Garai S, Sinha A. Biomimetic nanocomposites of carboxymethyl cellulose–hydroxyapatite: Novel three dimensional load bearing bone grafts. Colloids Surf B. 2014;115:182–90. 10.1016/j.colsurfb.2013.11.042 24342800

[39] Sundarrajan P, et al. One pot synthesis and characterization of alginate stabilized semiconductor nanoparticles. Bull Korean Chem Soc. 2012;33(10):3218–24.

[40] Huang A, et al. Synthesis and characterization of mesoporous hydroxyapatite powder by microemulsion technique. J Market Res. 2019;8(3):3158–66.

[41] Platero E, et al. Graphene oxide/alginate beads as adsorbents: Influence of the load and the drying method on their physicochemical-mechanical properties and adsorptive performance. J Colloid Interface Sci. 2017;491:1–12. 28011399 10.1016/j.jcis.2016.12.014

[42] Yang X, et al. Removal of Mn (II) by sodium alginate/graphene oxide composite double-network hydrogel beads from aqueous solutions. Sci Rep. 2018;8(1):1–16. 30013177 10.1038/s41598-018-29133-y PMC6048064

[43] Li Z, et al. Fabrication and evaluation of alginate/bacterial cellulose nanocrystals–chitosan–gelatin composite scaffolds. Molecules. 2021;26(16):5003. 34443588 10.3390/molecules26165003 PMC8400783

[44] Fan L, Zhang J, Wang A. In situ generation of sodium alginate/hydroxyapatite/halloysite nanotubes nanocomposite hydrogel beads as drug-controlled release matrices. J Mater Chem B. 2013;1(45):6261–70. 32261699 10.1039/c3tb20971g

[45] Khan MUA, et al. Graphene oxide-functionalized bacterial cellulose–gelatin hydrogel with curcumin release and kinetics: in vitro biological evaluation. ACS Omega. 2023;8(43):40024–35. 37929099 10.1021/acsomega.2c06825 PMC10620874

[46] Luz EPCG, et al. In vitro degradability and bioactivity of oxidized bacterial cellulose-hydroxyapatite composites. Carbohyd Polym. 2020;237: 116174. 10.1016/j.carbpol.2020.116174 32241452

[47] Dorozhkin SV. Nanodimensional and nanocrystalline apatites and other calcium orthophosphates in biomedical engineering, biology and medicine. Materials. 2009;2(4):1975–2045. 10.3390/ma9090752 PMC5457100 28773871

[48] da Rocha DN, et al. Mg substituted apatite coating from alkali conversion of acidic calcium phosphate. Mater Sci Eng C. 2017;70:408–17. 10.1016/j.msec.2016.09.021 27770910

[49] Lopes CC, et al. Nanocomposite powders of hydroxyapatite-graphene oxide for biological applications. Ceram Int. 2021;47(6):7653–65.

[50] Duan P, et al. Biomimetic mineralization and cytocompatibility of nanorod hydroxyapatite/graphene oxide composites. Front Chem Sci Eng. 2018;12:798–805.

[51] Ferraris S, et al. Bioactive materials: In vitro investigation of different mechanisms of hydroxyapatite precipitation. Acta Biomater. 2020;102:468–80. 31734414 10.1016/j.actbio.2019.11.024

